# Marrow angiogenesis-associated factors as prognostic biomarkers in patients with acute myelogenous leukaemia

**DOI:** 10.1038/sj.bjc.6603966

**Published:** 2007-09-11

**Authors:** C-Y Lee, H-F Tien, C-Y Hu, W-C Chou, L-I Lin

**Affiliations:** 1 Department of Clinical Laboratory Sciences and Medical Biotechnology, College of Medicine, National Taiwan University, No.1 Chang-Te Street, Taipei 10048, Taiwan; 2 Department of Internal Medicine, National Taiwan University Hospital, No.7 Chung Shan South Road, Taipei 10002, Taiwan; 3 Department of Laboratory Medicine, National Taiwan University Hospital, No.7 Chung Shan South Road, Taipei 10002, Taiwan

**Keywords:** prognostic biomarker, AML, angiopoietin family, VEGF family

## Abstract

Bone marrow (BM) neoangiogenesis plays an important role in acute myelogenous leukaemia (AML), and depends on the interplay of members of the vascular endothelial growth factor (VEGF) and angiopoietin (Ang) families. We determined the marrow levels of seven molecules associated with angiogenesis in 52 AML patients before chemotherapy and 20 healthy controls: VEGF-A, VEGF/PlGF, VEGF-C, VEGF-D, Ang-1, Ang-2, and Tie-2. All the molecules were quantified using enzyme-linked immunosorbent assay (ELISA). Comparing to normal controls, the marrow levels of VEGF/PlGF, Ang-2, and Tie-2 were significantly higher, and those of VEGF-C and Ang-1 were significantly lower in the AML patients (*P*<0.001). A total of 31 patients were further subjected to survival analysis. Patients with lower Tie-2 (<26 ng ml^−1^) and Ang-2 levels (<4500 pg ml^−1^) displayed a survival advantage (*P*=0.037 and 0.042, respectively), same as patients with higher VEGF/PlGF (⩾1 pg ml^−1^) and VEGF-D levels (⩾350 pg ml^−1^) (*P*=0.020 and 0.016, respectively). An angio-index ((Ang-2 × Tie-2)/(VEGF/PlGF × VEGF-D)) was established and multivariate Cox regression analysis revealed that patients with higher angio-index values (⩾50) displayed poor prognosis (hazard ratio 5.91, 95% confidence interval 1.99–17.56; *P*=0.001). The angio-index is closely associated with the clinical outcome of AML patients and may be valuable in disease prognosis.

Vascular endothelial growth factor (VEGF) and angiopoietin (Ang) are the two families involved in the regulation of vascular angiogenesis. The VEGF family comprises six members: VEGF-A, -B, -C, -D, -E, and placental growth factor (PlGF). They share three receptors, ie VEGFR-1, -2, and -3 (Korpelainen and Alitalo, 1998; Podar and Anderson, 2005). The Ang family includes four members: Ang-1, -2, -3, and -4. However, they share only a single receptor, ie Tie-2 (Ward and Dumont, 2002).

Both *in vitro* and *in vivo* investigations have revealed that VEGF-A was present in a high concentration in the bone marrow (BM) cells of AML patients and the vast majority of myeloblasts (>90%) could synthesise and secrete VEGF-A (Ghannadan *et al*, 2003). Compared to the levels in the control, increased plasma levels of VEGF-A obtained from peripheral blood (PB) were observed in AML patients (Aguayo *et al*, 2000, 2002). Elevated VEGF-C expression was reported in patients with prostatic carcinoma and oesophageal squamous cell carcinoma (Tsurusaki *et al*, 1999; Kitadai *et al*, 2001) but is controversial with regard to AML patients (Fielder *et al*, 1997; Dias *et al*, 2002; Loges *et al*, 2005). In contrast, decreased VEGF-D expression levels were reported in patients with lung adenocarcinoma and colorectal carcinoma (Niki *et al*, 2000; Onogawa *et al*, 2004). However, no previous reports addressed plasma VEGF-C and -D levels.

Both Ang-1 and -2 can only bind to the Tie-2 receptor, but they have opposite effects on Tie-2 activation. Tie-2 is a receptor tyrosine kinase that is expressed on endothelial cells (ECs) and haematopoietic stem cells (HSCs). Ang-1 functions as a stabilising signal for mature vasculature, and Ang-2 can be regarded as a regulator of vessel plasticity (Jones, 2003). In the BM niche, Ang-1/Tie-2 signalling occurs during interaction between Tie-2-expressing HSCs and Ang-1-expressing osteoblasts (Arai *et al*, 2004; Arai *et al*, 2005); this maintains the HSCs in the quiescence and anti-apoptotic state. Data from recent studies indicate that Ang-2 expression represents an independent prognostic factor in AML (Loges *et al*, 2005). However, the limitation of these studies was that plasma samples of the study patients were not available; thus, the investigation was restricted to the analysis of the mRNA expression of angiogenic factors. Therefore, precise data on the protein concentrations are essential.

Neoangiogenesis depends on the interplay of different members of the VEGF and Ang family, and many of these factors that are present in significant amounts in the marrow environment may function in a synchronised fashion in addition to any local autocrine or paracrine effects they may have; therefore, comprehensive analyses on marrow levels of these factors in AML patients and the clinical implications of these levels are important and remain to be explored.

## MATERIALS AND METHODS

### Study subjects

Prior to chemotherapy, marrow samples were obtained from 52 newly diagnosed AML patients at National Taiwan University Hospital (Taipei, Taiwan) from 1998 to 1999. This study was approved by the Institutional Review Board of the hospital (serial no 9461712129), and written informed consent was obtained from all patients. The study included 33 males and 19 females. The ages of the patients ranged from 2 to 90 years, with a median age of 45 years. According to the French–American–British (FAB) classification, two patients were classified as M0; 15, M1; 17, M2; 7, M3; 8, M4; 2, M5; and 1, unclassified. Of these, 14 patients did not undergo chemotherapy or were only treated with low-dose cytosine arabinoside due to old age and/or poor performance status; all the other patients with non-M3 AML subtypes underwent conventional induction chemotherapy with one of the anthracyclines (doxorubicin or idarubicin) for 3 days and cytosine arabinoside for 7 days. The patients with acute promyelocytic leukaemia (subtype M3) received all-trans retinoic acid with or without concurrent induction chemotherapy. After the patients achieved complete remission (CR), they underwent consolidation chemotherapy with a conventional dose of cytosine arabinoside and one anthracycline or with high-dose cytosine arabinoside. On the basis of tri-lineage cell regeneration with <5% blasts in the BM, normalisation of the PB cell count and the absence of leukaemia infiltration in the tissue, the patients were considered to have achieved CR. In addition, 20 healthy marrow donors were included as healthy controls in this study; of these, 10 were males and 7 females. The age of the control patients ranged from 11 to 56 years, with a median age of 32 years. After collection, the marrow samples were immediately centrifuged, and the plasma was separated and stored at −20 °C until investigation.

### Enzyme-linked immunosorbent assay

One-step sandwich enzyme immunoassay was performed to measure the VEGF-A, VEGF/PlGF, VEGF-C, VEGF-D, Ang-1, Ang-2, and Tie-2 concentrations by using commercially available kits (R&D systems, Minneapolis, MN, USA) according to the manufacturer's instructions.

The results represent the mean values of the duplicate determinations and were calculated using the SoftMax Pro 4.8 software (Molecular Devices Corporation, Sunnyvale, CA, USA).

### Statistical methods

Student's *t*-test and analysis of variance (ANOVA) were used to analyse the angiogenesis factors in the age, sex, FAB subtype, and karyotype subgroups of the AML patients. Spearman's test was used to analyse the correlation between the factors. The receiver operating characteristic (ROC) curve was constructed, and the area under the ROC curve (AUROC) was calculated for each factor to differentiate the patients from the controls. The paired *t*-test was used for patients who achieved CR to determine the difference between the concentrations of the various factors at presentation and CR. Survival status was investigated by using the Kaplan–Meier survival curve and log-rank test. Univariate and multivariate Cox regression analyses were also used to estimate prognosis. All the statistical analyses were performed by using the MedCalc software (http://www.medcalc.be/) and the SPSS 12.0 statistical software (SPSS Inc., Chicago, IL, USA).

## RESULTS

### Lack of correlation between plasma levels of seven angiogenesis-related factors in marrow and the egress of leukaemic cells from the BM to PB

The seven angiogenesis-related factors (VEGF-A, VEGF/PlGF, VEGF-C, VEGF-D, Ang-1, Ang-2, and Tie-2) in 52 AML patients at presentation were investigated, and no significant differences were observed in the age and FAB subtypes (Table 1). However, Tie-2 level was higher in males than that in females (*P*=0.024), and the Ang-1 level was significantly different among the karyotype subgroups (*P*=0.026).

To clarify whether these seven molecules present in the marrow microenvironment could determine the extent of egress of immature haematopoietic cells from the BM to PB, we correlated the absolute number of immature cells in the PB with the marrow levels of these molecules at disease presentation. We found that the marrow levels did not correlate with the egress of leukaemic cells from the BM to PB (VEGF-A, *r*=−0.050; VEGF/PlGF, *r*=0.229; VEGF-C, *r*=0.229; VEGF-D, *r*=0.014; Ang-1, *r*=0.406; Ang-2, *r*=0.169; and Tie-2, *r*=0.516).

### Significant difference in the levels of VEGF/PlGF, VEGF-C, Ang-1, Ang-2, and Tie-2 between healthy controls and AML patients

**Figure 1:**
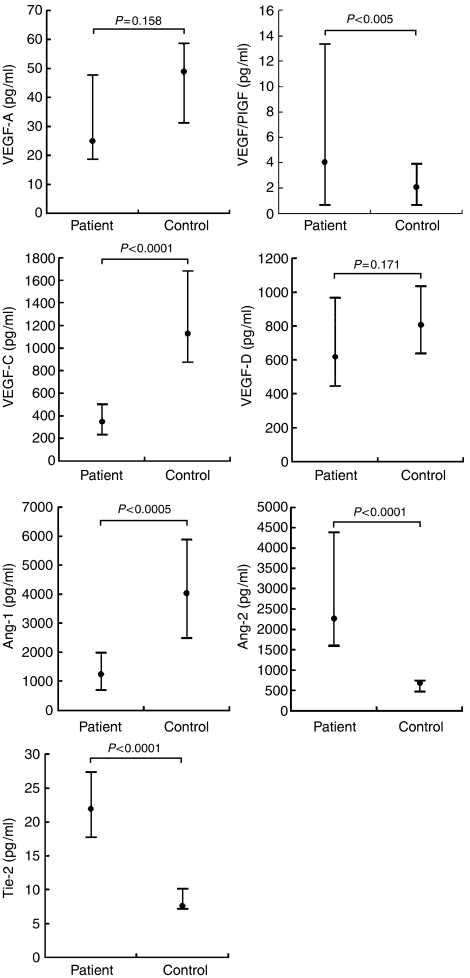
Marrow levels of vascular endothelial growth factor (VEGF)-A, VEGF/PlGF, VEGF-C, VEGF-D, angiopoietin (Ang)-1, Ang-2, and Tie-2 in 52 AML patients at presentation and in 20 healthy controls. Lower and upper lines indicate the 25th and 75th percentiles, respectively. The dot in each bar denotes the median. The *P*-value was calculated using the Student's *t*-test for the AML patient and control groups.

**Figure 2:**
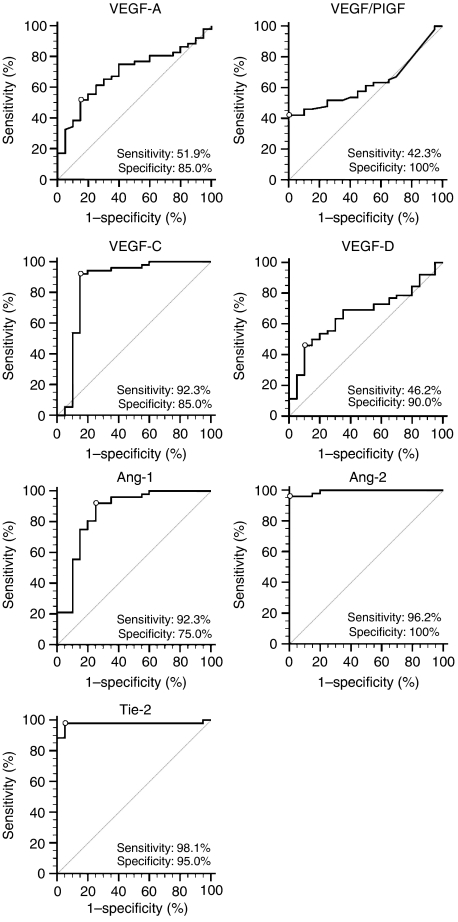
ROC curve of the angiogenesis factors in 52 AML patients at presentation and in 20 healthy controls. The white dots are the cut-off values between the patients and controls: vascular endothelial growth factor (VEGF)-A, 27.00 pg ml^−1^; VEGF/PlGF, 5.87 pg ml^−1^; VEGF-C, 664.20 pg ml^−1^; Ang-1, 2556.46 pg ml^−1^; Ang-2, 1039.77 pg ml^−1^; and Tie-2, 11.48 ng ml^−1^.

The marrow levels of these seven factors in the 52 AML patients at presentation were compared to those observed in the 20 healthy controls (Figure 1). Significant differences were observed in marrow levels of VEGF/PlGF, VEGF-C, Ang-1, Ang-2, and Tie-2 (*P*<0.005, *P*<0.0001, *P*<0.0005, *P*<0.0001, and *P*<0.0001, respectively). The ROC curve illustrated the cut-off value for each factor from which we could differentiate AML patients from the healthy controls (Figure 2). Among the seven factors, Ang-2 and Tie-2 displayed excellent results, with cut-off values of 1039.77 pg ml^−1^ and 11.48 ng ml^−1^, respectively. The AUROC was 0.993 (95% confidence interval (CI), 0.937–0.995; *P*<0.001) and 0.977 (0.910–0.997; *P*<0.001), respectively. VEGF-C and Ang-1 displayed good results, with cut-off values of 664.20 and 2556.46 pg ml^−1^, respectively, and the AUROC for these was 0.859 (0.756–0.929, *P*<0.001) and 0.861 (0.759–0.931, *P*<0.001), respectively. The remaining three molecules, ie VEGF-A, VEGF/PlGF, and VEGF-D displayed poor results, with an AUROC of 0.693 (0.573–0.796, *P*=0.009), 0.631 (0.509–0.742, *P*=0.063), and 0.667 (0.546–0.774, *P*=0.025), respectively.

### Sequential analysis of VEGF/PlGF, Ang-2, Tie-2, and VEGF-C levels in AML patients during the chemotherapy course

**Figure 3:**
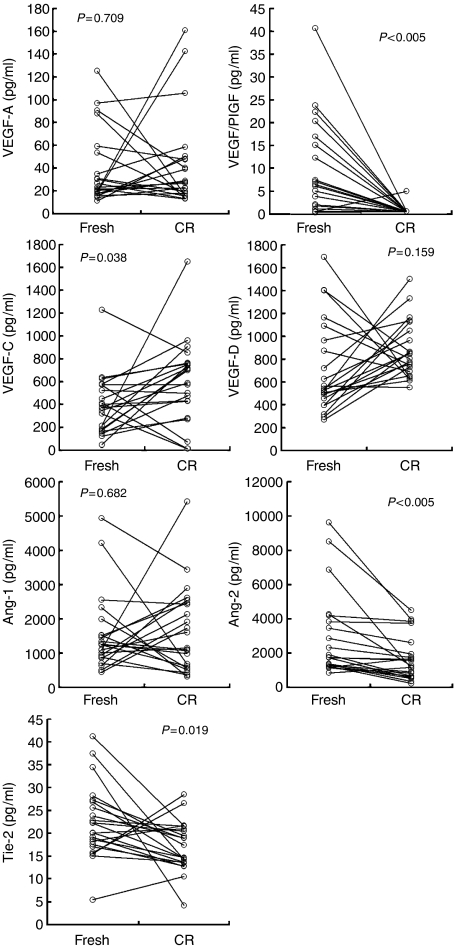
Levels of the angiogenesis factors in 22 patients at diagnosis and at complete remission (CR). Concentrations at the newly diagnosed (fresh) and first CR stages were compared. The *P*-value was calculated using the paired *t*-test.

Of the 31 AML patients undergoing conventional induction chemotherapy, 22 (71.0%) patients achieved CR after treatment. The marrow levels of VEGF/PlGF, Ang-2, Tie-2, and VEGF-C in the AML patients at presentation were compared to those observed at the CR stage. VEGF/PlGF and Ang-2 levels increased, while Tie-2 and VEGF-C levels decreased significantly (*P*<0.005, *P*<0.005, *P*=0.019, and *P*=0.038, respectively). All of these values were trend to normalise at CR stage as we expected, but they did not fit into normal range (Figure 3).

### Correlation of marrow levels of VEGF/PlGF, VEGF-D, and Tie-2 with prognosis of AML

**Figure 4:**
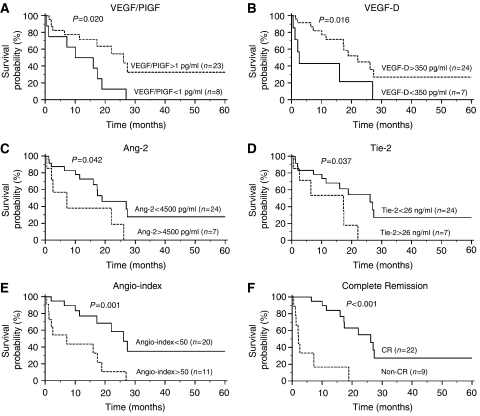
Kaplan–Meier survival curves for the angiogenesis factors, VEGF/PIGF (**A**), VEGF-D (**B**), Ang-2 (**C**), and Tie-2 (**D**), as well as angio-index (**E**) and complete remission achievement (**F**). Each angiogenesis factor was divided into high (dot line) and low (solid line) concentration subgroups based on the distribution of its level. The angio-index was divided into high (dot line) and low (solid line) subgroups with a cut-off value of 50. The *P*-value was calculated using the log-rank test. The angio-index stands for the formula (Ang-2 × Tie-2)(VEGF/PIGF × VEGF-D).

Further investigation was performed by using the Kaplan–Meier survival curve and log-rank test to evaluate the suitability of these molecules as prognostic factors. Of 31 AML patients undergoing conventional induction chemotherapy were divided into subgroups with high and low levels based on their distributions. The median follow-up period of the 31 patients was 12.0 months (range, 0.5–66.1 months). A 5-year survival analysis revealed significant differences in VEGF/PlGF, VEGF-D, Ang-2, and Tie-2 levels among the subgroups (Figure 4). Groups that demonstrated marrow VEGF/PlGF levels higher than 1 pg ml^−1^ or VEGF-D levels higher than 350 pg ml^−1^ demonstrated a greater probability of survival (*P*=0.020, 13.0 *vs* 26.2 months for VEGF/PlGF and *P*=0.016, 2.5 *vs* 22.1 months for VEGF-D). However, groups with marrow Tie-2 levels higher than 26 ng ml^−1^ or Ang-2 levels higher than 4500 pg ml^−1^ demonstrated a poor prognosis (*P*=0.037, 26.2 *vs* 17.3 months for Tie-2 and *P*=0.042, 18.9 *vs* 7.2 months for Ang-2). The remaining angiogenesis factors did not reveal any effect on overall survival. Based on the above results, we devised an algorithm known as the angio-index that collectively considered four distinct markers: (Ang-2 in pg ml^−1^ × Tie-2 in ng ml^−1^)/(VEGF/PlGF in pg ml^−1^ × VEGF-D in pg ml^−1^). We discovered that subgroups with an angio-index greater than 50 had substantially poor survival probabilities (*P*=0.001, 7.2 *vs* 26.2 months; Figure 4). In addition, we also analysed the 5-year survival of patients whether or not they achieved CR after standard chemotherapy, and discovered that the subgroups that achieved CR had a considerably better probability of survival (*P*<0.001, 26.2 *vs* 2.0 months; Figure 4). By comparing the probability of CR and the angio-index at diagnosis, we found that 18 of 20 (90%) patients with an angio-index less than 50 achieved CR after standard treatment; this suggests that the angio-index might be an appropriate indicator for the prediction of disease outcome.

In addition, by using the univariate Cox regression model, the angio-index displayed prominent prognosis value, with a hazard ratio of 4.38 (95% CI, 1.65–11.60, *P*=0.003); after adjusting for age and sex in a multivariate model, the hazard ratio may reach 5.91 (1.99–17.56, *P*=0.001).

## DISCUSSION

Marrow neoangiogenesis in AML is a complex process involving the interplay of different angiogenic growth factors. Until now, most investigators have attempted to elucidate the impact of a single angiogenic factor on the pathogenesis or prognosis of haematologic malignancies (Aguayo *et al*, 2002; Litwin *et al*, 2002; Moehler *et al*, 2003; Liu *et al*, 2005; Nowicki *et al*, 2006). However, HSCs were observed to be in close contact with the marrow stromal cells *in vivo* (Oostendorp and Dormer, 1997; Tordjman *et al*, 1999). Furthermore, both fibroblasts and microvascular ECs obtained in normal and leukaemic marrows were reported to produce several angiogenesis molecules (Moehler *et al*, 2003; De Raeve *et al*, 2004; Ogawa *et al*, 2006). Therefore, because of the synchronised action of various angiogenic cytokines, it is important to elucidate the marrow levels of the various angiogenesis factors present in the marrow microenvironment. The current study is the first report on the concentrations of the various angiogenesis factors in the marrow microenvironment of AML patients. We found that the marrow levels of VEGF/PlGF, Ang-2, and Tie-2 were significantly higher (*P*<0.001) and those of VEGF-C and Ang-1 were significantly lower (*P*<0.001) in the AML patients as compared to those values in healthy controls. In addition, we found that patients with lower VEGF/PlGF and VEGF-D levels or higher Ang-2 and Tie-2 levels displayed poor prognosis (*P*=0.020, 0.016, 0.042, and 0.037, respectively). We used the algorithm angio-index ((Ang-2 × Tie-2)/(VEGF/PlGF × VEGF-D)) and achieved the most accurate separation of patients with a high-risk signature from those with a low-risk signature (*P*=0.001). Further, the AML patients with higher angio-index values displayed poor prognosis, a hazard ratio of 4.38; suggesting that the combined marrow levels of VEGF/PlGF, VEGF-D, Ang-2, and Tie-2 facilitated prediction of prognosis. To our knowledge, this is the first report that evaluated prognosis by devising an algorithm that combined four distinct angiogenesis-associated factors.

The receptor tyrosine kinase Tie-2 is expressed in the ECs, early HSCs, and the so-called leukaemic cells (Arai *et al*, 2004; Arai *et al*, 2005). Ang-1, but not Ang-2, induces autophosphorylation of the Tie-2 receptor upon binding (Korpelainen and Alitalo, 1998; Jones, 2003; Thurston, 2003; Peters *et al*, 2004). Therefore, Ang-1 and Ang-2 are believed to function as naturally occurring antagonists. The cellular receptor Tie-2 shedding from the cell surface results in the release of a soluble receptor from Tie-2-expressing cells (Reusch *et al*, 2001). Consequently, the soluble receptor can be detected in human serum and plasma. Recently, Schliemann *et al* (2006) used an immunohistochemical stain to study BM biopsy samples and discovered that both Ang-2 and Tie-2 were overexpressed in the leukaemic blasts of AML patients; however, Ang-1 was not Loges *et al* (2005) employed quantitative RT–PCR to study mRNA expression and discovered that the median levels of VEGF-A, VEGF-C, Ang-1, and Ang-2 were higher in PB blasts than in normal PB AC133^+^ cells. In our study, we also found a similar trend for Ang-2 and Tie-2 in the AML patients; nevertheless, we further revealed significant differences in the Ang-1, VEGF/PlGF, and VEGF-C levels between the AML patients and healthy controls. These reports demonstrated that the Ang-2 and Tie-2 levels displayed a similar trend in AML patients with regard to cellular mRNA and proteins and extracellular proteins. However, similar trends were not observed for VEGF-A, VEGF-C, and Ang-1 levels; this suggests that the distribution of three angiogenesis-associated factors in the cells and microenvironment may vary, and while some display a positive association, others display a negative association. Therefore, the molecules in intracellular and extracellular environments exhibited different implications in prognosis prediction. Schliemann *et al* (2006) discovered that patients expressing high intracellular Ang-2 levels in leukaemic cells displayed a significantly extended overall survival than that displayed by patients with low intracellular Ang-2 levels Loges *et al* (2005) discovered that higher Ang-2 mRNA levels in PB blasts tend to indicate a better prognosis in AML patients. However, in our study, high marrow levels of extracellular Ang-2 indicated a poor prognosis. This diversity might be from different sample origin, one was from PB and the other was from marrow.

In the angiogenesis process, VEGF-A is usually present in its homodimeric form. It can also form a heterodimer with PlGF, and this stimulates angiogenesis via the VEGFR-1/VEGFR-2 heterodimer (Autiero *et al*, 2003; Roy *et al*, 2006); this is consistent with our finding that VEGF/PlGF was present at substantially higher levels in AML patients than in the healthy controls. Loges *et al* (2005) reported that the AML patients and healthy donors demonstrated no significant difference in the level of VEGF-C mRNA expression. Fielder *et al* (1997) reported that leukaemic cells expressed VEGF-C in most of the AML patients. Dias *et al* (2002) found that VEGF-C that was released from the endothelium could induce leukaemic cell proliferation, promote their survival, and protect them from chemotherapy-induced apoptosis. In our study, we observed a difference in the marrow VEGF-C levels between the AML patients and healthy controls, but we could not predict the prognosis of the AML patients based on these levels (*P*=0.591).

Our previous report (Lin *et al*, 2002) addressed the close relationship between the marrow matrix metalloproteinase (MMP)-9 level and the disease status of AML; the marrow MMP-9 level may be a useful surrogate marker for monitoring the disease status in AML patients. However, the survival times of AML patients with lower MMP-9 levels were only slightly longer than those of AML patients with higher levels (*P*=0.12). As observed in this report, the angio-index involving four angiopoiesis-associated factors was an appropriate prognostic marker (*P*=0.001). Therefore, the estimation of the marrow MMP-9, VEGF/PlGF, VEGF-D, Ang-2, and Tie-2 levels may be helpful in predicting the disease status of AML patients. Further prospective and multi-centre studies would be worthwhile to verify the wide application of these biomarkers.

## Figures and Tables

**Table 1:** Characteristics of the study groups

	**No.**	**VEGF (pg ml^−1^)**	**VEGF/PlGF (pg ml^−1^)**	**VEGF-C (pg ml^−1^)**	**VEGF-D (pg ml^−1^)**	**Ang-1 (pg ml^−1^)**	**Ang-2 (pg ml^−1^)**	**Tie-2 (ng ml^−1^)**
*Age*								
<45	25	47.85±43.85	8.52±10.63	378.89±258.09	614.55±381.32	1344.02±939.19	3977.02±2741.20	23.89±8.15
⩾45	27	31.49±19.37	19.02±37.01	389.53±247.86	879.69±582.55	1509.71±939.30	2936.34±2742.42	21.81±7.60
*P*-value		0.096	0.168	0.880	0.057	0.528	0.178	0.346
								
*Sex*								
Male	33	41.40±36.56	15.45±31.14	391.74±251.72	844.10±560.19	1279.31±800.31	3192.22±2513.06	24.52±8.36
Female	19	35.81±30.03	11.41±21.78	371.70±254.39	592.64±366.79	1691.86±1103.48	3861.23±3183.72	19.83±6.03
*P*-value		0.554	0.587	0.785	0.057	0.164	0.438	0.024^a^
								
*FAB subtype*								
M0	2	44.13±35.22	9.09±9.20	282.84±21.65	829.40±517.51	1725.07±915.97	1213.66±585.51	24.32±0.74
M1	15	35.48±44.46	3.39±3.68	348.72±186.96	764.07±703.42	1480.47±659.10	3283.47±2327.49	23.40±5.71
M2	17	37.55±25.65	26.76±40.63	417.03±338.62	726.52±462.79	1735.12±1350.26	3362.76±3219.54	20.18±8.67
M3	7	71.88±37.89	7.80±10.92	413.84±131.58	687.09±279.06	783.75±269.15	3951.64±3160.14	24.29±3.90
M4	8	26.14±14.28	17.32±31.84	459.35±263.12	667.53±321.93	1464.84±440.60	4131.93±3134.01	24.75±8.85
M5	2	20.11±1.35	4.35±5.22	89.07±10.65	820.14±272.82	545.18±137.94	3460.07±1308.26	28.80±24.15
*P*-value		0.133	0.275	0.505	0.996	0.211	0.855	0.583
								
*Cytogenetic subtype*								
Favourable	7	55.14±45.01	8.44±10.51	369.97±159.36	702.40±271.53	988.34±848.59	2952.35±2516.61	21.87±5.73
Intermediate	29	37.08±37.03	18.48±35.32	426.92±301.19	726.98±445.84	1735.36±1029.81	3588.60±3129.72	24.55±9.18
Unfavourable	16	36.58±21.30	8.22±13.77	313.69±157.26	819.76±688.43	1069.93±535.84	3373.17±2240.56	20.07±5.06
*P*-value		0.429	0.434	0.351	0.817	0.026^a^	0.861	0.179

a Denotes significant difference.

Abbreviations: Ang=angiopoietin; ANOVA=analysis of variance; FAB=French–American–British; PlGF=placental growth factor; VEGF=vascular endothelial growth factor.

Age and sex were analysed using Student's *t* test, and FAB subtypes and cytogenetic subtype were analysed using ANOVA.

Favourable cytogenetic subtype: t(15; 17), t(8; 21), and inv(16); unfavourable: −5, −7, +8, and complex; and intermediate: normal cytogenetic subtype and others.

## References

[bib1] Aguayo A, Kantarjian H, Manshouri T, Gidel C, Estey E, Thomas D, Koller C, Estrov Z, O'Brien S, Keating M, Freireich E, Albitar M (2000) Angiogenesis in acute and chronic leukemias and myelodysplastic syndromes. Blood 96: 2240–2245 10979972

[bib2] Aguayo A, Kantarjian HM, Estey EH, Giles FJ, Verstovsek S, Manshouri T, Gidel C, O'Brien S, Keating MJ, Albitar M (2002) Plasma vascular endothelial growth factor levels have prognostic significance in patients with acute myeloid leukemia but not in patients with myelodysplastic syndromes. Cancer 95: 1923–1930 12404286 10.1002/cncr.10900

[bib3] Arai F, Hirao A, Ohmura M, Sato H, Matsuoka S, Takubo K, Ito K, Koh GY, Suda T (2004) Tie2/angiopoietin-1 signaling regulates hematopoietic stem cell quiescence in the bone marrow niche. Cell 118: 149–161 15260986 10.1016/j.cell.2004.07.004

[bib4] Arai F, Hirao A, Suda T (2005) Regulation of hematopoietic stem cells by the niche. Trends Cardiovasc Med 15: 75–79 15885574 10.1016/j.tcm.2005.03.002

[bib5] Autiero M, Waltenberger J, Communi D, Kranz A, Moons L, Lambrechts D, Kroll J, Plaisance S, De Mol M, Bono F, Kliche S, Fellbrich G, Ballmer-Hofer K, Maglione D, Mayr-Beyrle U, Dewerchin M, Dombrowski S, Stanimirovic D, Van Hummelen P, Dehio C, Hicklin DJ, Persico G, Herbert JM, Communi D, Shibuya M, Collen D, Conway EM, Carmeliet P (2003) Role of PlGF in the intra- and intermolecular cross talk between the VEGF receptors Flt1 and Flk1. Nat Med 9: 936–943 12796773 10.1038/nm884

[bib6] De Raeve H, Van Marck E, Van Camp B, Vanderkerken K (2004) Angiogenesis and the role of bone marrow endothelial cells in haematological malignancies. Histol Histopathol 19: 935–950 15168356 10.14670/HH-19.935

[bib7] Dias S, Choy M, Alitalo K, Rafii S (2002) Vascular endothelial growth factor (VEGF)-C signaling through FLT-4 (VEGFR-3) mediates leukemic cell proliferation, survival, and resistance to chemotherapy. Blood 99: 2179–2184 11877295 10.1182/blood.v99.6.2179

[bib8] Fielder W, Graeven U, Ergun S, Verago S, Kilic N, Stockschlader M, Hossfeld DK (1997) Expression of FLT4 and its ligand VEGF-C in acute myeloid leukemia. Leukemia 11: 1234–1237 9264375 10.1038/sj.leu.2400722

[bib9] Ghannadan M, Wimazal F, Simonitsch I, Sperr WR, Mayerhofer M, Sillaber C, Hauswirth AW, Gadner H, Chott A, Horny HP, Lechner K, Valent P (2003) Immunohistochemical detection of VEGF in the bone marrow of patients with acute myeloid leukemia—correlation between VEGF expression and the FAB category. Am J Clin Pathol 119: 663–671 12760284 10.1309/331Q-X7AX-KWFJ-FKXM

[bib10] Jones PF (2003) Not just angiogenesis—wider roles for the angiopoietins. J Pathol 201: 515–527 14648654 10.1002/path.1452

[bib11] Kitadai Y, Amioka T, Haruma K, Tanaka S, Yoshihara M, Sumii K, Matsutani N, Yasui W, Chayama K (2001) Clinicopathological significance of vascular endothelial growth factor (VEGF)-C in human esophageal squamous cell carcinomas. Int J Cancer 93: 662–666 11477575 10.1002/ijc.1379

[bib12] Korpelainen EI, Alitalo K (1998) Signaling angiogenesis and lymphangiogenesis. Curr Opin Cell Biol 10: 159–164 9561839 10.1016/s0955-0674(98)80137-3

[bib13] Lin LI, Lin DT, Chang CJ, Lee CY, Tang JL, Tien HF (2002) Marrow matrix metalloproteinases (MMPs) and tissue inhibitors of MMP in acute leukaemia: potential role of MMP-9 as a surrogate marker to monitor leukaemic status in patients with acute myelogenous leukaemia. Br J Haematol 117: 835–841 12060118 10.1046/j.1365-2141.2002.03510.x

[bib14] Litwin C, Leong KG, Zapf R, Sutherland H, Naiman SC, Karsan A (2002) Role of the microenvironment in promoting angiogenesis in acute myeloid leukemia. Am J Hematol 70: 22–30 11994978 10.1002/ajh.10092

[bib15] Liu P, Li JY, Han ZC, Lu H, Wang Y, Xu B, Peng Z (2005) Elevated plasma levels of vascular endothelial growth factor is associated with marked splenomegaly in chronic myeloid leukemia. Leuk Lymphoma 46: 1761–1764 16263579 10.1080/10428190500262318

[bib16] Loges S, Heil G, Bruweleit M, Schoder V, Butzal M, Fischer U, Gehling UM, Schuch G, Hossfeld DK, Fiedler W (2005) Analysis of concerted expression of angiogenic growth factors in acute myeloid leukemia: expression of angiopoietin-2 represents an independent prognostic factor for overall survival. J Clin Oncol 23: 1109–1117 15718307 10.1200/JCO.2005.05.058

[bib17] Moehler TM, Ho AD, Goldschmidt H, Barlogie B (2003) Angiogenesis in hematologic malignancies. Crit Rev Oncol Hematol 45: 227–244 12633837 10.1016/s1040-8428(02)00135-x

[bib18] Niki T, Iba S, Tokunou M, Yamada T, Matsuno Y, Hirohashi S (2000) Expression of vascular endothelial growth factors A, B, C, and D and their relationships to lymph node status in lung adenocarcinoma. Clin Cancer Res 6: 2431–2439 10873096

[bib19] Nowicki M, Ostalska-Nowicka D, Kaczmarek E, Miskowiak B, Witt M (2006) Vascular endothelial growth factor C—a potent risk factor in childhood acute lymphoblastic leukaemia: an immunocytochemical approach. Histopathology 49: 170–177 16879394 10.1111/j.1365-2559.2006.02465.x

[bib20] Ogawa M, LaRue AC, Drake CJ (2006) Hematopoietic origin of fibroblasts/myofibroblasts: its pathophysiologic implications. Blood 108: 2893–2896 16840726 10.1182/blood-2006-04-016600

[bib21] Onogawa S, Kitadai Y, Tanaka S, Kuwai T, Kimura S, Chayama K (2004) Expression of VEGF-C and VEGF-D at the invasive edge correlates with lymph node metastasis and prognosis of patients with colorectal carcinoma. Cancer Sci 95: 32–39 14720324 10.1111/j.1349-7006.2004.tb03167.x PMC11159672

[bib22] Oostendorp RAJ, Dormer P (1997) VLA-4-mediated interactions between normal human hematopoietic progenitors and stromal cells. Leuk Lymphoma 24: 423–435 9086434 10.3109/10428199709055581

[bib23] Peters KG, Kontos CD, Lin PC, Wong AL, Rao P, Huang LW, Dewhirst MW, Sankar S (2004) Functional significance of Tie2 signaling in the adult vasculature. Recent Prog Horm Res 59: 51–71 14749497 10.1210/rp.59.1.51

[bib24] Podar K, Anderson KC (2005) The pathophysiologic role of VEGF in hematologic malignancies: therapeutic implications. Blood 105: 1383–1395 15471951 10.1182/blood-2004-07-2909

[bib25] Reusch P, Barleon B, Weindel K, Martiny-Baron G, Godde A, Siemeister G, Marme D (2001) Identification of a soluble form of the angiopoietin receptor TIE-2 released from endothelial cell and present in human blood. Angiogenesis 4: 123–131 11806244 10.1023/a:1012226627813

[bib26] Roy H, Bhardwaj S, Yla-Herttuala S (2006) Biology of vascular endothelial growth factors. Febs Lett 580: 2879–2887 16631753 10.1016/j.febslet.2006.03.087

[bib27] Schliemann C, Bieker R, Padro T, Kessler T, Hintelmann H, Buchner T, Berdel WE, Mesters RM (2006) Expression of angiopoietins and their receptor Tie2 in the bone marrow of patients with acute myeloid leukemia. Haematologica 91: 1203–1211 16956819

[bib28] Thurston G (2003) Role of angiopoietins and Tie receptor tyrosine kinases in angiogenesis and lymphangiogenesis. Cell Tissue Res 314: 61–68 12915980 10.1007/s00441-003-0749-6

[bib29] Tordjman R, Ortega N, Coulombel L, Plouet J, Romeo PH, Lemarchandel V (1999) Neuropilin-1 is expressed on bone marrow stromal cells: a novel interaction with hematopoietic cells? Blood 94: 2301–2309 10498602

[bib30] Tsurusaki T, Kanda S, Sakai H, Kanetake H, Saito Y, Alitalo K, Koji T (1999) Vascular endothelial growth factor-C expression in human prostatic carcinoma and its relationship to lymph node metastasis. Br J Cancer 80: 309–313 10390013 10.1038/sj.bjc.6690356 PMC2362987

[bib31] Ward NL, Dumont DJ (2002) The angiopoietins and Tie2/Tek: adding to the complexity of cardiovascular development. Semin Cell Dev Biol 13: 19–27 11969368 10.1006/scdb.2001.0288

